# Algorithmic Bias in AI-Based Diagnostic Models: A Systematic Review and Lifecycle Framework for Fairness Auditing and Governance in Healthcare

**DOI:** 10.3390/healthcare14193293

**Published:** 2026-10-02

**Authors:** Reyadh Alluhaibi

**Affiliations:** Department of Artificial Intelligence and Data Science, College of Computer Science and Engineering, Taibah University, Medina 42353, Saudi Arabia; rluhaibi@taibahu.edu.sa

**Keywords:** algorithmic bias, artificial intelligence in healthcare, health equity, clinical governance, responsible AI, systematic review, bias mitigation, fairness auditing, post-deployment monitoring, medical imaging

## Abstract

**Background:** Artificial-intelligence-based diagnostic systems increasingly influence healthcare delivery, yet strong aggregate performance can coexist with clinically meaningful disparities across demographic subgroups. Such disparities raise not only model-development concerns but also health-equity, implementation, and governance challenges for healthcare organizations. **Methods:** We conducted a systematic review reported in accordance with PRISMA 2020 of literature published from January 2019 through August 2026. The search identified 1842 records; 87 records were retained in the overall evidence corpus and 46 underwent structured quantitative extraction. The corpus included primary empirical/model-evaluation evidence together with secondary or adjacent methodological evidence used for qualitative contextualization. We also developed FRCS-10, a ten-item descriptive reporting-completeness index derived from established reporting and governance guidance, and applied it to the full corpus. **Results:** Peer-reviewed primary sources reported baseline true-positive-rate gaps of 0.062–0.281 across sex, age, and race on CheXpert and MIMIC-CXR, while a contextual dermatology meta-analysis reported AUROC values of 0.89 for Fitzpatrick I–III and 0.82 for Fitzpatrick IV–VI. These values were synthesized as source-reported estimates and were not independently re-analysed from patient-level data. Data representation was the most frequently coded bias source (38%), followed by algorithmic design (21%) and label or annotation bias (19%). The descriptive FRCS-10 mean was 4.6/10; only 7 of 87 records described post-deployment fairness monitoring. Five primary empirical studies with sufficiently explicit estimands were assessed for compatibility; their disparity definitions were not commensurable, so no pooled effect size was computed. **Conclusions:** We propose the Equity-Aware Bias Detection and Mitigation Framework (EA-BDMF), a literature-derived, implementation-oriented lifecycle specification for healthcare AI. It links data auditing, prevalence-aware preprocessing, fairness-constrained training, calibration and threshold governance, and post-deployment monitoring. The framework is accompanied by a Lifecycle Equity Debt (LED) accounting measure, checkpoint documentation, a Bias Provenance–Mitigation Traceability Matrix, and descriptive regulatory mapping. The complete framework has not yet been validated end to end, and its clinical and organizational utility requires prospective evaluation in real-world healthcare settings.

## 1. Introduction

Artificial intelligence (AI) is increasingly embedded in healthcare delivery, including radiological triage, dermatological screening, sepsis prediction, clinical risk stratification, and decision-support workflows [1,2]. As these systems move from retrospective model development into routine care, their consequences are no longer limited to technical performance: they affect who receives timely detection, how clinical resources are prioritized, and whether existing inequities are reproduced through digital infrastructure. Aggregate discrimination metrics can conceal systematic differences in model behavior across sex, age, race, ethnicity, and skin tone [3,4,5]. A model may therefore retain an acceptable overall AUROC while producing substantially different false-negative or true-positive rates for specific subgroups [4,6].

The problem is well documented in medical imaging. Studies using MIMIC-CXR and CheXpert have reported materially higher underdiagnosis or lower sensitivity for Black patients, women, and younger patients, with reported true-positive-rate (TPR) gaps exceeding 0.28 for race in some cohorts [3,6]. Dermatology presents a related representation problem. Datasets and models dominated by lighter skin tones have produced lower diagnostic performance for darker skin, and a recent meta-analysis reported an AUROC gap of 0.07 between Fitzpatrick I–III and IV–VI groups [7,8,9]. These findings illustrate a broader implementation problem: fairness failures can emerge from data composition, label construction, model optimization, evaluation design, threshold selection, workflow integration, and post-deployment distribution shift [1,2].

For healthcare organizations, the practical challenge is consequently broader than selecting a debiasing algorithm. Fairness must be translated into auditable decisions about dataset governance, model development, calibration, deployment thresholds, monitoring responsibilities, and remediation when subgroup performance deteriorates. This lifecycle perspective is increasingly reflected in reporting and governance initiatives such as TRIPOD+AI, STANDING Together, and FUTURE-AI, as well as in emerging regulatory expectations for risk management, human oversight, and post-market monitoring [10,11,12,13,14,15]. Yet technical fairness studies and healthcare-governance guidance remain only weakly connected in operational terms.

The literature has responded along three largely separate lines. Clinical and governance reviews characterize bias across the AI lifecycle but generally stop short of specifying an auditable mitigation workflow [1,2]. Fairness-methodology studies provide a large set of mathematical criteria, many of which are mutually incompatible under unequal prevalence and only weakly connected to clinical utility [16,17,18,19]. A third body of work evaluates individual interventions—including resampling, adversarial debiasing, counterfactual augmentation, pruning, and adapter retraining—on heterogeneous datasets and outcome definitions [20,21,22]. Because these studies do not share a common evaluation harness, their reported effects cannot be interpreted as a controlled comparison of mitigation strategies or directly translated into an organizational deployment policy.

This fragmentation creates two related gaps. First, the evidence base does not provide an integrated, auditable link between empirically reported bias sources, mitigation actions, deployment decisions, monitoring, and organizational accountability. Second, the literature lacks a common reporting substrate for cumulative quantitative synthesis: subgroup sample sizes, uncertainty estimates, decision thresholds, demographic-metadata provenance, and post-deployment monitoring are often incompletely reported. The present study addresses these gaps by integrating systematic evidence synthesis, an explicit estimand-compatibility assessment, a descriptive reporting-completeness instrument, and a stage-resolved governance specification that makes residual disparity, evidence provenance, and unresolved mitigation gaps visible across the AI lifecycle.

The study has four objectives. First, we characterize the magnitude and type of demographic disparities reported for diagnostic AI on reproducible public benchmarks. Second, we identify where bias is most frequently reported to enter the diagnostic-AI lifecycle and summarize the evidence for major mitigation families. Third, we quantify reporting completeness using FRCS-10 and determine whether the extracted literature supports a defensible cross-study effect-size synthesis. Fourth, we propose EA-BDMF, an auditable five-stage framework that connects technical mitigation with healthcare governance, residual-disparity accounting, traceability, and longitudinal monitoring. The framework contributes three formal artifacts: (i) Lifecycle Equity Debt (LED), defined below as a tolerance-exceedance measure across model-bearing checkpoints; (ii) a Bias Provenance–Mitigation Traceability Matrix that exposes coverage gaps between reported bias sources and validated interventions; and (iii) FRCS-10, a structured reporting-completeness instrument derived from PRISMA 2020, TRIPOD+AI, STANDING Together, and FUTURE-AI [10,11,12,23]. We additionally map the framework to potentially applicable provisions of Regulation (EU) 2024/1689, FDA lifecycle guidance, and 45 C.F.R. §92.210 [13,14,15]. Together, these elements position fairness as a healthcare quality and governance problem that must remain observable after deployment, not merely as a model-development objective.

## 2. Related Work

This section situates the proposed framework within the broader literature on fairness, implementation, and governance in healthcare AI. It first examines how fairness is conceptualized across the healthcare AI lifecycle and reviews the principal mathematical definitions used to characterize subgroup disparities. It then summarizes empirical evidence of demographic performance differences in diagnostic applications, with particular attention to medical imaging, before reviewing mitigation strategies, fairness auditing, and post-deployment monitoring. Together, these strands establish the conceptual and empirical context for linking model-level fairness methods with the organizational requirements of safe, equitable, and accountable healthcare implementation.

### 2.1. Lifecycle Fairness and Fairness Definitions

Healthcare-AI fairness is increasingly treated as a lifecycle property rather than a one-time validation criterion. Bias can originate in problem formulation, cohort construction, label definition, model development, evaluation, implementation, and post-deployment feedback loops [1,2]. Recent guidance reflects this lifecycle view. FUTURE-AI frames fairness, robustness, traceability, and usability as requirements for trustworthy deployment [12]; STANDING Together focuses on transparent dataset documentation [11]; TRIPOD+AI strengthens prediction-model reporting [10]; and CLAIM and DECIDE-AI provide complementary guidance for medical-imaging AI and early clinical evaluation [24,25].

The mathematical fairness literature adds an important constraint: fairness is not a single optimization target. Group criteria such as independence, separation, sufficiency, equal opportunity, and calibration encode different normative choices [17]. Under unequal base rates, calibration and error-rate balance cannot generally be satisfied simultaneously [18,19]. A clinical framework should therefore make the selected fairness target, tolerance, and decision threshold explicit rather than treating them as implicit implementation details.

### 2.2. Empirical Disparities in Diagnostic AI

Chest-radiograph studies using MIMIC-CXR and CheXpert have repeatedly documented subgroup differences in sensitivity and underdiagnosis [6,26]. Foundation-model evaluations have further shown that disparities can become more pronounced at intersections of protected attributes, including among Black female patients [4]. Representation analyses indicate that demographic information can remain strongly encoded in learned features even when protected attributes are not supplied explicitly [6]. Dataset audits also document label noise, demographic imbalance, and domain shift in widely used radiography benchmarks [27].

Dermatology shows a similar interaction between representation and measurement. The Diverse Dermatology Images (DDI) dataset was created to improve tonal diversity, yet remains small relative to large radiology corpora [28]. The construct used to encode skin tone is itself consequential: Fitzpatrick Skin Type was not designed as a general skin-tone scale, and comparative work has questioned its validity and repeatability for AI fairness evaluation [29,30]. These concerns are amplified by evidence that generative image systems can underrepresent darker skin tones or produce diagnostically unreliable synthetic dermatologic images [31,32].

### 2.3. Mitigation, Auditing, and Post-Deployment Monitoring

Bias mitigation is commonly organized into pre-processing, in-processing, and post-processing families. Pre-processing approaches include resampling and reweighting; prevalence-aware oversampling has produced large relative reductions in race-associated false-negative-rate disparity in chest radiography [3]. In-processing approaches include adversarial representation learning and multi-attribute fairness objectives [33,34,35]. Post-processing approaches include threshold selection, calibration, and lightweight adapter retraining [22,36,37]. Counterfactual augmentation and bias-aware pruning extend this space but have been evaluated under different protocols and cannot be ranked directly against the preceding methods [20,21].

Recent work also shifts attention from static validation to auditing and maintenance. FAMEWS integrates subgroup metrics, prevalence-aware event measures, statistical testing, and bias-source checks in a reproducible fairness audit [38]. Post-deployment monitoring has been formulated as a sequential testing problem in which repeated evaluation must control false-alarm rates [39]. Longitudinal evidence further suggests that calibration and operational drift can emerge before delayed outcome-based discrimination metrics reveal degradation [40]. These findings motivate an explicit monitoring stage rather than treating fairness as fixed at release.

## 3. Materials and Methods

This section describes the methodological procedures used to identify, select, appraise, and synthesize the evidence on algorithmic bias in AI-based diagnostic models. It first defines the review design, eligibility criteria, and database search strategy, then details the study-selection and data-extraction procedures. The section subsequently introduces the FRCS-10 reporting-completeness instrument and concludes by specifying the evidence-synthesis approach, the benchmark datasets represented in the included literature, and the fairness metrics used throughout the analysis.

### 3.1. Review Design, Eligibility, and Search Strategy

The review was conducted and reported in accordance with PRISMA 2020 and its explanation and elaboration document [23,41]. The review covered literature published from January 2019 through August 2026. The completed PRISMA 2020 checklist is provided as Supplementary File S1.

Primary empirical and model-evaluation studies were eligible when they examined algorithmic bias, fairness, or bias-mitigation methods in AI-based diagnostic or clinical-prediction models and reported subgroup-stratified performance for at least one demographic or phenotypic attribute. Peer-reviewed journal articles, conference papers, and indexed preprints were eligible.

Secondary and methodological publications, including systematic or scoping reviews, critical appraisals, tutorials, and closely related studies of bias mechanisms, were also retained when they directly informed fairness definitions, bias provenance, mitigation strategies, reporting practices, or governance. These sources were used to support the qualitative synthesis and conceptual development of the review, but were not treated as independent primary estimates in cross-study quantitative comparisons.

When a peer-reviewed version of an eligible preprint was available before the 20 August 2026 search cutoff, that version was used as the primary citation. Two of the 87 included records (2.3%) were available only as preprints at the search cutoff. Supplementary Table S2 reports the evidence role and publication status of each included record, and Supplementary Table S4 summarizes publication status across the corpus.

We excluded studies that did not report demographic or phenotypic subgroup analyses, non-English publications, opinion or perspective articles without an empirical or systematic methodological basis, and studies based exclusively on proprietary datasets when subgroup findings could not be related to a publicly documented benchmark or otherwise interpreted with sufficient methodological transparency. This criterion was intended to ensure that the quantitative evidence included in the synthesis could be traced to sufficiently documented data and evaluation procedures. PubMed/MEDLINE, IEEE Xplore, the ACM Digital Library, Scopus, Web of Science, and arXiv (cs.LG, cs.CY, and eess.IV) were searched on 20 August 2026. The core Boolean expression was

(“algorithmic bias” OR “fairness” OR “disparity”) AND (“artificial intelligence” OR “machine learning” OR “deep learning”) AND (“diagnosis” OR “diagnostic” OR “clinical prediction”) AND (“demographic” OR “race” OR “sex” OR “age” OR “skin tone”).

The query syntax was adapted to the indexing conventions of each database, including controlled-vocabulary terms where available. The review was not prospectively registered in PROSPERO or another public registry, and no formal review protocol was publicly deposited before screening. This limits comparison of the final eligibility and synthesis decisions with a prospectively time-stamped analysis plan, as discussed in Section 7.1.

### 3.2. Study Selection and Data Extraction

Title and abstract screening was performed by the author using the predefined eligibility criteria and was not independently duplicated. Full-text eligibility was independently assessed by the author and an external assessor using the same criteria. Disagreements were resolved through discussion, with unresolved cases referred to a third independent assessor. Inter-rater agreement for full-text eligibility decisions was assessed using Cohen’s κ and was 0.81.

The search identified 1842 records. After removal of 527 duplicates, 1315 records underwent title/abstract screening, of which 1041 were excluded. A total of 274 reports were sought for retrieval; 24 could not be retrieved, leaving 250 reports for full-text eligibility assessment. Of these, 163 were excluded: 92 because results were not stratified by demographic or phenotypic attributes, 51 because the study addressed a non-diagnostic clinical task, and 20 because the publication was not in English. Eighty-seven records were retained for the review.

Structured quantitative extraction was undertaken for 46 of the 87 included records. These comprised 41 non-secondary empirical or related records and five secondary or contextual sources used to support interpretation rather than as independent primary effect estimates. The quantitative-extraction subset therefore represented 52.9% of the included records and 2.5% of the 1842 records initially identified.

**Figure 1:**
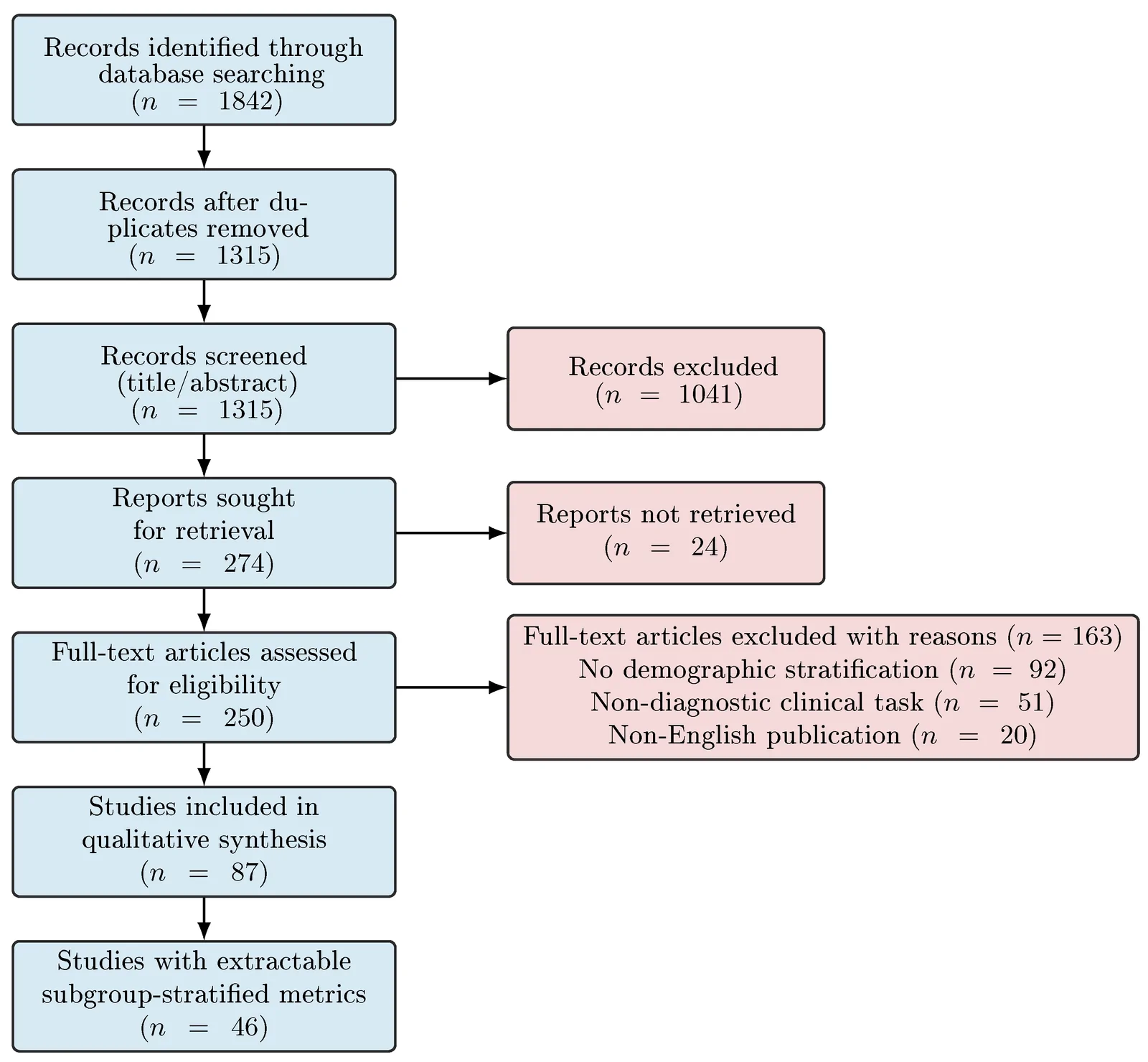
PRISMA 2020 flow diagram showing record identification, screening, eligibility assessment, and study inclusion.

The study-selection process is summarized in Figure 1, and report-level screening dispositions are provided in Supplementary Table S3.

Data extraction was performed by the author using a predefined structured form and was not independently duplicated. Extracted variables included study design, dataset, demographic attributes, reported fairness metrics, mitigation strategy, subgroup sample sizes where available, uncertainty estimates, decision thresholds, and effect estimates.

A review-specific overall methodological appraisal was recorded for all 87 included records. For primary prediction-model evidence, the appraisal drew on the four PROBAST domains—Participants, Predictors, Outcome, and Analysis—and adapted them to the scope of this review. The Participants domain considered data-source appropriateness, selection, and subgroup inclusion; Predictors considered definition, measurement consistency, timing, and potential leakage; Outcome considered outcome definition, reference-standard credibility, and consistency of ascertainment; and Analysis considered sample size, missing data, split independence, overfitting, performance estimation, threshold specification where relevant, and uncertainty in subgroup comparisons.

An overall Low/Moderate/High appraisal was recorded for each of the 87 included records, yielding 24 Low, 48 Moderate, and 15 High classifications. For secondary and contextual publications, formal PROBAST application was not appropriate; their ratings were therefore treated as review-specific overall methodological appraisals. The additional Moderate category denotes material but interpretable concerns and is not an official PROBAST category. Supplementary Table S1 provides the 87 study-specific overall appraisals and FRCS-10 totals, the mapping to PROBAST/PROBAST+AI concepts, and detailed domain-level examples for the principal empirical quantitative studies. The appraisal process was performed by the author and was not independently duplicated.

### 3.3. FRCS-10 Reporting-Completeness Instrument

Risk-of-bias tools evaluate the credibility of estimates; they do not directly measure whether a fairness study reports the information required for interpretation, replication, or cross-study synthesis. We therefore constructed FRCS-10, a ten-item Fairness Reporting Completeness Score. Candidate items were derived from PRISMA 2020, TRIPOD+AI, STANDING Together, and FUTURE-AI [10,11,12,23], then consolidated by operational equivalence. Patient-level splitting and post-deployment fairness monitoring were retained as separate items because the auditing literature identifies both as consequential for leakage control and lifecycle surveillance [38,39,40].

Each item receives 1 point when explicitly reported and 0 otherwise, yielding a total score from 0 to 10. Items are equally weighted because no empirical basis currently supports differential weighting. FRCS-10 was scored by the author and independently verified by a second assessor; discrepancies were resolved by consensus, with third-assessor adjudication when necessary.

FRCS-10 was used as a descriptive measure of reporting completeness rather than as an assessment of methodological quality, risk of bias, or clinical validity. It was applied to all 87 included records. For secondary or contextual publications, model-development-specific items received credit only when explicitly reported, and lower totals were not interpreted as evidence of poorer study quality. FRCS-10 scores were not used to determine study inclusion, methodological-appraisal classification, or eligibility for quantitative synthesis.

### 3.4. Bias-Source Coding

Bias sources were coded at the level of distinct reported mechanisms rather than at the study level. A single study could therefore contribute more than one coded instance. The coding framework was organized around five recurring sources of bias in the healthcare-fairness literature: data representation, algorithmic design, label and annotation processes, deployment and feedback, and evaluation and reporting. Conceptually similar mechanisms were grouped within the same category according to their underlying source.

Data representation covered sampling, prevalence, demographic coverage, and related composition effects; algorithmic design covered mechanisms arising from model learning, representation, or optimization; label and annotation covered target definition, annotator effects, proxy labels, and related outcome-construction mechanisms; deployment and feedback covered distribution shift and workflow-mediated effects; and evaluation and reporting covered incomplete subgroup evaluation or insufficient documentation. Percentages were calculated using the total number of coded bias instances as the denominator and represent reporting frequencies rather than study prevalence or estimates of causal contribution. Supplementary Table S4 provides the operational definitions and coding rules.

### 3.5. Evidence Synthesis, Datasets, and Metrics

Qualitative findings were synthesized thematically because the included literature differed substantially in clinical task, subgroup definition, fairness metric, decision threshold, and mitigation strategy. Quantitative subgroup or fairness information was suitable for structured extraction in 46 records, comprising 41 non-secondary records and five secondary/contextual records. Quantitative information from secondary or contextual sources was used to support interpretation but was not treated as an independent primary effect in cross-study comparisons.

All 46 quantitatively extracted records were examined for synthesis suitability. Twelve records met a reporting-completeness criterion requiring subgroup sample sizes, uncertainty estimates, and an explicit decision threshold when a thresholded estimand was reported. This criterion assessed the interpretability of reported quantitative results rather than eligibility for meta-analysis.

A separate estimand-compatibility assessment was conducted for five primary empirical studies in which the reported contrast and evaluation context were sufficiently specified to identify the statistical target. These studies differed in subgroup contrast, performance functional, thresholding convention, mitigation estimand, and evaluation context, and therefore did not estimate a sufficiently common statistical parameter for pooling. In particular, TPR gaps, false-negative-rate differences, AUROC differences, distributional comparisons, and benchmark accuracy were not transformed onto a common effect-size scale because they represent distinct statistical estimands. Supplementary Table S4 summarizes the quantitative-synthesis criteria and the study-specific reasons why pooling was not performed.

All numerical disparity estimates reported below are source-reported values attributed to independent published studies using publicly accessible, de-identified datasets. Patient-level predictions were not obtained for independent recomputation; the quantitative synthesis therefore used published aggregate results and focused on their consistency, interpretation, and comparability. Table 1 summarizes the principal benchmark resources represented in the synthesis.

The reviewed literature most commonly reports AUROC, F1-score, subgroup TPR/FNR, Statistical Parity Difference, and AUROC parity. For a binary sensitive attribute $A\in \{a_{0},a_{1}\}$, we report the magnitude of the Equal Opportunity Difference as the absolute TPR gap:

(1) $$\mathrm{EOD}=\left|\mathrm{TPR}\left(A=a_{0}\right)-\mathrm{TPR}\left(A=a_{1}\right)\right|.$$

This EOD magnitude equals zero under exact TPR parity; the absolute-value convention is stated explicitly because signed definitions are also used in the fairness literature. Because fairness criteria encode different normative and statistical assumptions, all extracted values are reported on the scale defined by their source study rather than re-expressed as if they measured a single latent construct [16,17].

Because the candidate studies did not share a common effect-size scale, no formal assessment of small-study effects or publication bias was performed. No formal certainty-of-evidence framework was applied because the review synthesized heterogeneous diagnostic-fairness estimands rather than a common intervention effect.

## 4. Results

This section presents the principal findings of the review and organizes them around the sources, reporting characteristics, and observed consequences of algorithmic bias in diagnostic AI. It first summarizes the taxonomy of bias mechanisms identified across the included studies, then evaluates the completeness of fairness-related reporting using FRCS-10. The section subsequently reports the baseline demographic disparities documented in the literature, reviews the effects of individual mitigation strategies on their source-defined metrics, and concludes with an estimand-compatibility assessment that determines whether a quantitative cross-study synthesis is methodologically defensible.

### 4.1. Bias Sources Identified in the Literature

**Figure 2:**
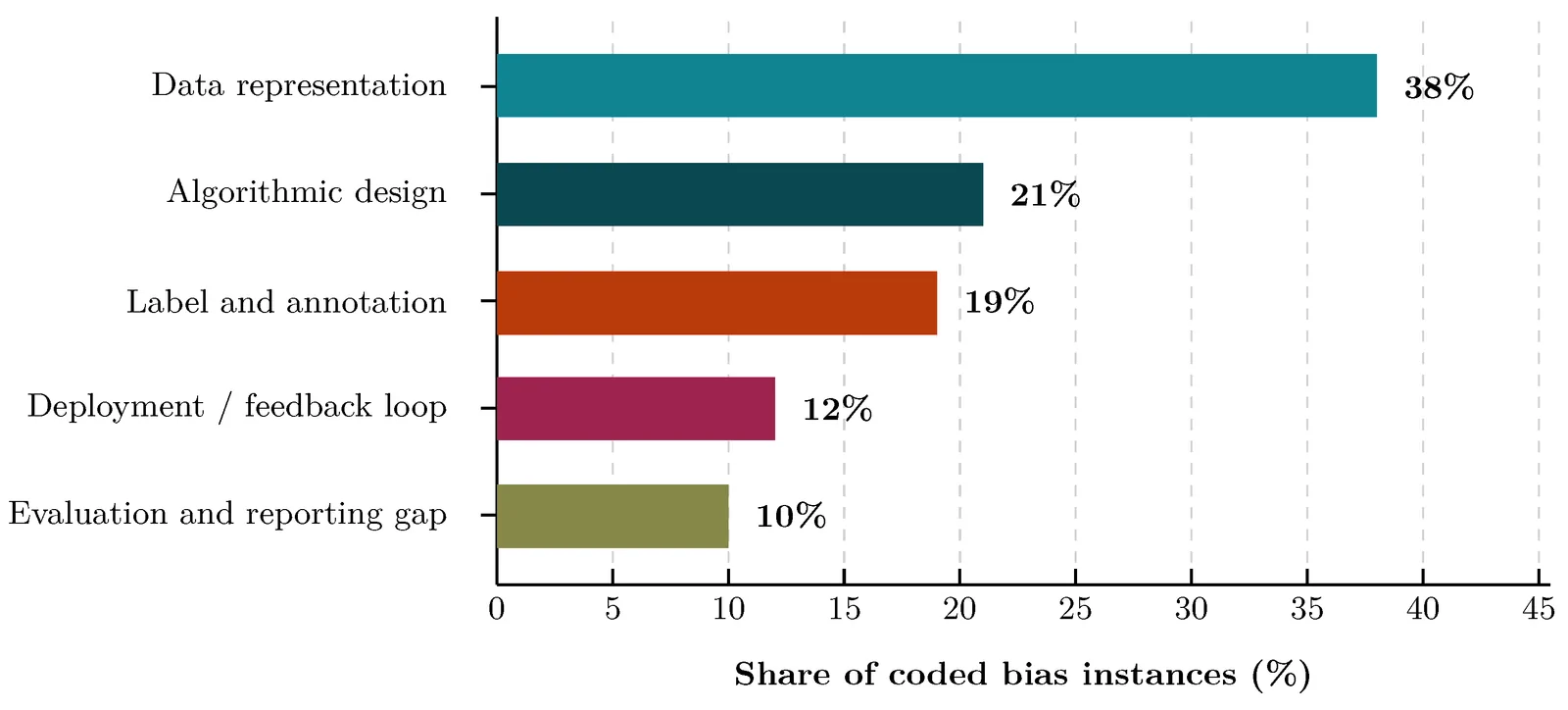
Distribution of coded bias sources across the included studies. Percentages represent the share of coded bias instances assigned to each bias-source category; individual studies could contribute to more than one category.

Thematic coding yielded five bias-source categories. Data representation was the most frequently coded category (38% of coded instances), followed by algorithmic design (21%), label and annotation bias (19%), deployment and feedback-loop effects (12%), and evaluation or reporting gaps (10%). The coding unit was a distinct bias instance rather than a study; one study could therefore contribute to more than one category. These percentages describe reporting frequency in the included corpus rather than a causal decomposition of observed performance disparity. Table 2 summarizes the mechanisms associated with each category, while Figure 2 visualizes their relative frequency in the coded corpus.

### 4.2. Fairness Reporting Completeness

FRCS-10 scores varied substantially across the 87 included records. The mean score was 4.6/10, with a median of 4 and an interquartile range of 3–6; five records reported at least 8 of the 10 prespecified items, and none reported all 10. These scores describe reporting completeness rather than methodological quality.

All 87 records identified the protected attributes analyzed, and 83% reported subgroup sample sizes. Reporting was less complete for elements needed to reproduce or compare subgroup estimates: demographic-metadata provenance was documented in 30% of records, uncertainty accompanied subgroup performance in 44%, the decision threshold and its selection procedure were reported in 36%, and patient-level rather than image-level splitting was explicitly confirmed in 47%. Intersectional analyses were reported in 22% of records, while 25% reported a mitigation effect together with its corresponding accuracy cost on the same cohort. Post-deployment or temporal fairness monitoring was reported in only 7 of 87 records (8%).

Item-level reporting completeness is summarized in Table 3, and study-level FRCS-10 totals are provided in Supplementary Table S1.

Of the 46 records with structured quantitative extraction, 12 met the reporting-completeness criterion requiring subgroup sample sizes, uncertainty, and an explicit threshold when a thresholded estimand was used. Estimand compatibility was assessed separately in five primary empirical studies with sufficiently explicit statistical targets and evaluation contexts (Section 4.5). Reporting completeness and estimand compatibility address distinct requirements for quantitative synthesis: a well-reported study may still be unsuitable for pooling when its statistical target or evaluation context is not commensurable with those of other studies.

### 4.3. Baseline Demographic Disparities

**Figure 3:**
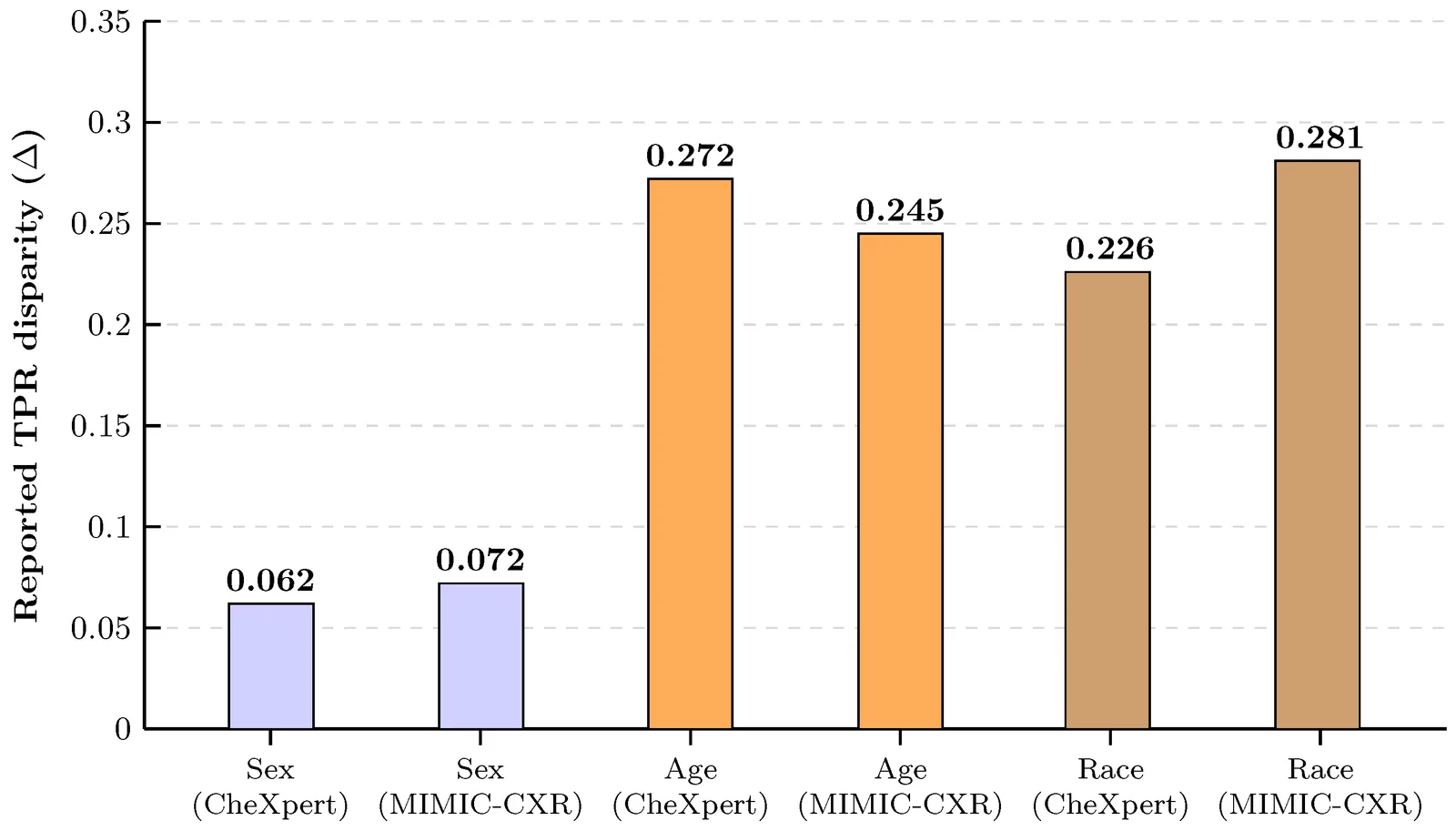
Reported true-positive-rate (TPR) disparities across sex, age, and race subgroups on CheXpert and MIMIC-CXR. Values are reproduced from the source study and represent source-specific subgroup TPR gaps.

Kurian et al. reported TPR gaps of 0.062 (sex, CheXpert), 0.072 (sex, MIMIC-CXR), 0.272 (age, CheXpert), 0.245 (age, MIMIC-CXR), 0.226 (race, CheXpert), and 0.281 (race, MIMIC-CXR) [6]. These values are shown in Figure 3 and Table 4. Patient-level predictions were not available for independent recalculation, so the values are presented as study-specific source-reported measurements.

In dermatology, Tjiu and Lu reported pooled AUROC values of 0.89 for Fitzpatrick I–III and 0.82 for Fitzpatrick IV–VI, corresponding to a gap of 0.07 [9]. This estimate comes from a separate meta-analysis and is not directly comparable with the TPR gaps above. It is also conditioned on Fitzpatrick annotation, whose validity as a proxy for skin tone has been questioned [29,30].

### 4.4. Evidence for Individual Mitigation Strategies

The mitigation literature supports the direction of several intervention effects, but not a cross-study ranking. Jackson, Yan, and Malin reported a 74.7% relative reduction in race-associated FNR disparity after prevalence-based oversampling, with no material change in AUC; synthetic augmentation in the same cohort produced a 10.6% relative reduction [3]. These two interventions are directly comparable because they share the same source study, cohort, model, and disparity definition. By contrast, adapter retraining, adversarial debiasing, and council-guided methods were evaluated under different metrics and protocols [22,33,47]. We therefore preserve source-native metrics and avoid constructing a synthetic “combined” mitigation effect. Table 5 summarizes the source-attributed intervention effects and the constraints on their cross-study comparability.

### 4.5. Estimand Compatibility and Quantitative Synthesis

Five studies initially appeared sufficiently close in imaging-based bias evaluation to merit explicit estimand comparison. Detailed inspection showed that they did not estimate the same statistical quantity. Seyyed-Kalantari et al. operationalized underdiagnosis through the false-positive rate of a binarized “no finding” label [26]; Kurian et al. reported multiple subgroup TPR gaps [6]; Jackson et al. reported a race-associated FNR difference and relative mitigation effects [3]; Yang et al. reported thresholded underdiagnosis/FNR disparities across demographic groups, with rank-based tests used for selected model–radiologist comparisons [4]; and Luo et al. reported benchmark accuracy under induced-bias scenarios [47]. These quantities differ in outcome orientation, subgroup contrast, pathology, thresholding, dataset, and comparator structure and therefore do not define a common study-level effect. The resulting estimand-compatibility assessment is summarized in Table 6.

No pooled effect size or forest plot was produced because the candidate estimands did not share a meaningful common scale. Computing *Q*, $I^{2}$, or a prediction interval across non-commensurable quantities would not yield a well-defined common parameter. The synthesis therefore preserves source-native metrics: TPR gaps are reported as TPR gaps, the dermatology AUROC difference is reported separately, and distributional tests are treated as significance evidence rather than converted into performance-gap estimates.

## 5. EA-BDMF: A Healthcare Lifecycle Framework for Fairness Auditing and Governance

**Figure 4:**
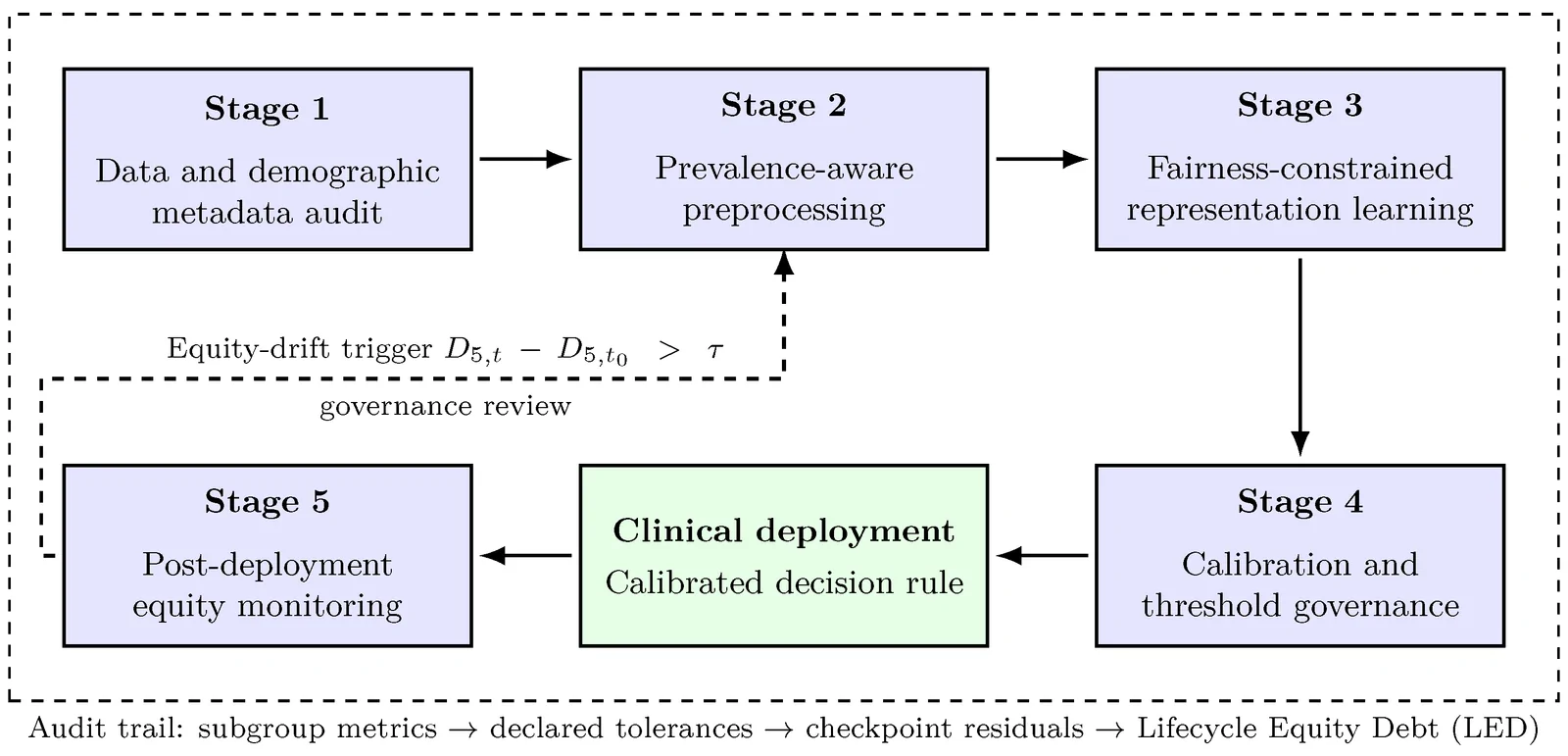
EA-BDMF lifecycle architecture. The framework separates data auditing, prevalence-aware preprocessing, fairness-constrained representation learning, calibration and threshold governance, and post-deployment equity monitoring. Clinical deployment occurs after calibration and governance review. A pre-specified equity-drift condition triggers reassessment of the preprocessing and subsequent model-development stages. The dashed enclosure denotes the audit trail used to document subgroup metrics, declared tolerances, checkpoint residuals, and Lifecycle Equity Debt (LED). Stage 1 produces data-governance artifacts but no model-performance residual; disparity-based checkpoint accounting begins only once an executable model is available.

EA-BDMF organizes the evidence above into five linked lifecycle stages (Figure 4) and is intended as an implementation-oriented governance specification for healthcare organizations. It identifies where independently studied mitigation families can act, the audit artifacts produced at each stage, how residual disparity can be tracked across model-bearing checkpoints, and where clinical, technical, and governance decisions constrain deployment options. The complete five-stage workflow has not yet been evaluated end to end; its current evidentiary basis is therefore component-level rather than pipeline-level.

### 5.1. Lifecycle Stages

Stage 1—Data and demographic-metadata audit.

Incoming datasets are audited for subgroup prevalence, missing demographic metadata, and annotation provenance [1,11]. When skin tone is in scope, the audit records the annotation instrument because Fitzpatrick Skin Type may be an imperfect proxy for skin tone [29,30]. The stage should also preserve label provenance and annotator-agreement information where available. If the audit identifies material label-integrity concerns, the workflow should pause before model optimization and require a documented label-quality remediation decision—for example, targeted re-annotation/adjudication, sensitivity analysis to alternative label definitions, or evaluation of a label-noise-robust training strategy. The reviewed diagnostic-AI fairness literature does not establish a preferred label-remediation strategy for this stage.

Stage 2—Prevalence-aware preprocessing.

The framework supports prevalence-aware resampling or reweighting and subgroup-restricted conventional augmentation. Evidence is strongest for oversampling in the reviewed chest-radiograph literature [3]. Synthetic image generation is not adopted as a default mitigation because dermatology studies have documented deficiencies in tonal diversity and diagnostic fidelity of generated images [31,32].

Stage 3—Fairness-constrained representation learning.

A protected-attribute adversary can be attached to the learned representation and trained through gradient reversal. For encoder parameters, the standard objective is

(2) $$L=L_{\mathrm{task}}-\lambda L_{\mathrm{adv}},$$

where λ controls the strength of the adversarial constraint. EA-BDMF does not prescribe a universal value for λ; any value used in an empirical implementation should be selected by a pre-specified validation procedure that reports both subgroup fairness and task-performance consequences on held-out data. A directly relevant diagnostic-imaging implementation is CLEAR-AI, which uses multibranch adversarial debiasing with dynamically weighted gradient reversal on chest radiographs and reports reductions in TPR and FPR disparities across multiple confounders without a measurable loss in task performance [48]. When several protected attributes are material, concurrent multi-attribute objectives are preferable to optimizing one attribute in isolation, because single-attribute optimization can leave or redistribute disparities across other protected dimensions [34,35].

Stage 4—Calibration and threshold governance.

Calibration is estimated on a held-out calibration set after model training has converged. Platt scaling is a parametric default and isotonic regression an alternative when calibration data are sufficiently large [36,37]. Subgroup-specific thresholds are not treated as an automatic deployment rule. They are a governance-gated option because selecting a threshold by protected-group membership can alter clinical decisions and may engage jurisdiction-specific legal and ethical constraints. If institutional review does not authorize subgroup-specific decision rules, the framework uses a shared threshold and records the remaining disparity rather than masking it.

Stage 5—Post-deployment equity monitoring.

Subgroup-stratified discrimination and calibration are monitored over time. Repeated testing should use a pre-specified error-control strategy rather than unbudgeted thresholding of rolling metrics [39]. Calibration drift is tracked alongside discrimination because it may emerge before delayed outcome labels reveal changes in AUROC or sensitivity [40]. A monitoring event that exceeds the pre-declared equity-drift threshold triggers reassessment of data composition and preprocessing rather than silent threshold adjustment. Table 7 summarizes the bias source addressed at each process stage, the corresponding methodological action, and the audit artifact or model-bearing checkpoint generated by that stage.

### 5.2. Lifecycle Equity Debt

Residual performance disparity can be compared across the lifecycle only at checkpoints where an executable model or decision rule exists. We therefore distinguish the five *process stages* of EA-BDMF from its *model-bearing audit checkpoints*. Stage 1 produces data-governance artifacts but no TPR-based debt, because no model prediction exists before training. Likewise, the effect of Stage 2 is evaluated only after an otherwise comparable model has been trained under the Stage 2 preprocessing policy. In a common-harness validation, a typical checkpoint set is $C=\{c_{0},c_{2},c_{3},c_{4}\}$, representing an untreated reference model, the model retrained after Stage 2 preprocessing, the Stage 3 fairness-constrained model, and the Stage 4 calibrated decision rule. Post-deployment windows generate additional time-indexed checkpoints $c_{5,t}$.

Let A denote the protected attributes under review and $G_{a}$ the groups defined for attribute *a*. For every checkpoint c∈C, let $M_{c}\left(g\right)$ denote the same clinically relevant performance functional—for example, TPR—evaluated for group *g* on a pre-declared, patient-disjoint audit set. For each attribute, a clinically justified tolerance $\varepsilon_{a}>0$ is fixed before inspecting the checkpoint results. The observed subgroup spread is

(3) $$\Delta_{c}\left(a\right)=\max_{g,g^{\prime }\in G_{a}}\left|M_{c}\left(g\right)-M_{c}\left(g^{\prime }\right)\right|.$$

The tolerance-exceedance debt for attribute *a* at checkpoint *c* is

(4) $$d_{c}\left(a\right)={\left[\frac{\Delta_{c}\left(a\right)}{\varepsilon_{a}}-1\right]}_{+}=\max\left\{0,\frac{\Delta_{c}\left(a\right)}{\varepsilon_{a}}-1\right\},$$

so that $d_{c}\left(a\right)=0$ exactly when the observed subgroup spread does not exceed the declared tolerance. The checkpoint residual is

(5) $$D_{c}=\frac{1}{\left|A\right|}\sum_{a\in A}d_{c}\left(a\right).$$

For a declared finite audit horizon C, Lifecycle Equity Debt is the weighted aggregate

(6) $${\mathrm{LED}}_{C}=\sum_{c\in C}w_{c}D_{c},\;w_{c}>0,\;\sum_{c\in C}w_{c}=1.$$

The checkpoint set, audit population, performance functional, tolerances, and weights are therefore part of the estimand and must be declared with the audit. LED is an accounting summary of tolerance exceedance, not a causal decomposition of bias. In particular, a change between two checkpoints localizes where the recorded disparity changed; it does not establish which upstream mechanism caused that change.

**Proposition** **1** (Checkpoint monotonicity)**.** *Let T be a mitigation operator introduced between two model-bearing checkpoints. If, for every protected attribute a and every checkpoint c downstream of T, the observed spread satisfies $\Delta_{c}^{T}\left(a\right)\leq \Delta_{c}\left(a\right)$, while upstream checkpoint values remain unchanged, then ${\mathrm{LED}}_{C}^{T}\leq {\mathrm{LED}}_{C}$. If the inequality is strict for at least one attribute at a checkpoint with positive weight and no other downstream spread increases, then ${\mathrm{LED}}_{C}^{T}<{\mathrm{LED}}_{C}$.*

**Proof.**  For every attribute *a*, the mapping $x\mapsto {\left[x/\varepsilon_{a}-1\right]}_{+}$ is non-decreasing for $\varepsilon_{a}>0$. Hence $\Delta_{c}^{T}\left(a\right)\leq \Delta_{c}\left(a\right)$ implies $d_{c}^{T}\left(a\right)\leq d_{c}\left(a\right)$. Averaging over attributes yields $D_{c}^{T}\leq D_{c}$ at every affected checkpoint, while upstream residuals are unchanged. Equation (6) is a positive weighted sum of checkpoint residuals, giving ${\mathrm{LED}}_{C}^{T}\leq {\mathrm{LED}}_{C}$. Strict decrease follows when at least one positively weighted checkpoint residual decreases strictly and none increases.    □

**Corollary** **1** (Auditable checkpoint certificates)**.** *Given the declared checkpoint set C and weights $\left\{w_{c}\right\}$, the vector $\left\{D_{c}:c\in C\right\}$ is sufficient to recompute ${\mathrm{LED}}_{C}$. To make each $D_{c}$ independently reproducible without disclosing patient-level records, a checkpoint certificate should report the protected-group definitions, evaluation-set identifier or version, decision threshold and calibration rule, subgroup denominators and the sufficient aggregate counts for the selected performance functional (for TPR, at minimum TP and FN), the tolerances $\varepsilon_{a}$, and the resulting $D_{c}$.*

**Remark** **1** (Interpretive limits)**.** *LED is not itself a fairness criterion. It inherits the selected performance functional, protected attributes, subgroup definitions, audit population, tolerances, checkpoint set, and weights. It cannot detect disparity associated with an unmeasured attribute or an intersection that was not pre-specified, and it does not resolve the incompatibility among calibration, error-rate parity, and other fairness definitions [18,19]. It must therefore be reported with the underlying subgroup-specific estimates and their uncertainty, never as a substitute for them.*

Proposition 1 establishes only an algebraic property of the declared accounting function: if all downstream measured spreads weakly decrease, the corresponding LED value cannot increase. It does not show that LED predicts patient harm, clinical utility, regulatory acceptability, or deployment outcomes. Those properties require prospective empirical validation and sensitivity analysis over the selected performance functional, tolerances, subgroup definitions, and checkpoint weights.

### 5.3. Illustrative Worked Example

An illustrative LED calculation can be constructed from the source-reported race-associated FNR disparity in Jackson et al. [3]. The reported baseline disparity was approximately 0.084, and prevalence-based oversampling reduced it by 74.7% in the same cohort, giving an illustrative post-mitigation disparity of approximately 0.0213. If an institution had prospectively declared a race-disparity tolerance of ε=0.05 and equal weights for the untreated and post-preprocessing checkpoints, the untreated checkpoint debt would be ${\left[0.084/0.05-1\right]}_{+}=0.68$, the post-preprocessing debt would be ${\left[0.0213/0.05-1\right]}_{+}=0$, and the two-checkpoint LED would be 0.34. The tolerance and checkpoint weights are hypothetical governance choices; only the baseline disparity and relative mitigation effect are source-reported. The example illustrates the LED calculation and its audit traceability. A step-by-step version is provided in Supplementary File S2.

### 5.4. Framework Orchestration and Equity-Drift Trigger

The orchestration distinguishes process artifacts from model-bearing audit checkpoints. A patient-disjoint audit set is fixed before model comparison; calibration is fitted only after representation learning has converged; and the calibration observations are not reused as the final audit set. This separation is necessary for the checkpoint quantities in Section 5.2 to have a stable interpretation. Algorithm 1 specifies the complete EA-BDMF workflow from data auditing through deployment-time equity monitoring.

The monitoring trigger is a governance rule rather than a universal statistical constant. The threshold τ must be pre-specified with reference to the sampling variability of subgroup estimates and the repeated-testing procedure used in Stage 5 [39]. Because deployment windows can differ in case mix from the development audit set, the time-indexed monitoring residual is compared with a defined deployment reference period rather than silently combined with development checkpoints.
**Algorithm 1** EA-BDMF orchestration with model-bearing equity-debt checkpoints.**Require:** Dataset *D*; protected attributes A; tolerances $\left\{\varepsilon_{a}\right\}$; checkpoint weights $\left\{w_{c}\right\}$; drift threshold τ**Ensure:** Deployed model and decision rule; data-audit artifacts; checkpoint certificates; LED trace 1: Create patient-disjoint training, validation, calibration, and audit partitions. 2: Audit demographic metadata, subgroup prevalence, label provenance, and annotation scheme. {Stage 1; no TPR-based LED checkpoint} 3: Train an untreated reference model under the declared architecture and evaluate checkpoint $c_{0}$ on the audit partition. 4: Apply the pre-specified prevalence-aware sampling/weighting and permitted augmentation policy. 5: Retrain the same reference architecture under the Stage 2 policy and evaluate checkpoint $c_{2}$. 6: Train the fairness-constrained model under Stage 3 and evaluate checkpoint $c_{3}$. 7: Fit calibration on the calibration partition. 8: **if** subgroup-specific thresholds receive institutional approval **then** 9:       Fit the approved subgroup-specific decision thresholds.10: **else**11:     Use a shared threshold and retain the residual disparity in the audit record.12: **end if**13: Evaluate the calibrated decision rule at checkpoint $c_{4}$ and compute ${\mathrm{LED}}_{C}$ over the declared development checkpoints.14: Deploy with a pre-specified monitoring window and sequential error-control policy.15: **for** each monitoring window *t* **do**16:     Compute subgroup metrics and the time-indexed residual $D_{5,t}$ using the same metric definitions.17:     **if** $D_{5,t}-D_{5,t_{0}}>\tau$ **then**18:         Trigger remediation and re-enter Stage 2 after governance review.19:     **end if**20: **end for**

### 5.5. Bias Provenance–Mitigation Traceability

Mapping the bias taxonomy to EA-BDMF reveals uneven mitigation coverage. Data-representation bias has direct support for data auditing and prevalence-aware preprocessing; algorithmic-design bias has direct support for fairness-aware training and post hoc correction. Deployment drift is addressable through monitoring, although prospective fairness-specific validation remains limited. Evaluation gaps are primarily addressed by instrumentation rather than by model optimization.

The clearest coverage gap concerns labels and annotations. This category accounts for 19% of coded bias instances, yet the reviewed diagnostic-AI fairness corpus contains no validated EA-BDMF component that repairs erroneous or proxy-substituted labels after they have entered the training data. Stage 1 therefore includes a label-quality remediation gate: a material provenance or agreement problem must be documented and resolved, or its sensitivity evaluated, before downstream model optimization proceeds. Re-annotation, adjudication, alternative label definitions, and label-noise-robust training are plausible actions, but the reviewed evidence does not establish a preferred method or a fairness benefit for the complete pipeline. Reweighting, representation learning, and threshold adjustment still condition on the supplied labels. This conclusion is limited to the diagnostic-AI fairness literature included in the review; label-noise-robust learning methods outside this corpus were not evaluated. The source-to-stage mapping and its corresponding audit artifacts are reported in Table 8.

### 5.6. Regulatory and Governance Mapping

The legal force of the instruments mapped to EA-BDMF is not uniform, and the mapping below is descriptive rather than a compliance determination. Applicability depends on the product classification, intended use, deployer/provider role, jurisdiction, institutional status, and the facts of a specific implementation. Healthcare organizations should therefore obtain appropriate legal and regulatory review rather than treating Table 9 as an operational compliance checklist. Regulation (EU) 2024/1689 is binding EU law, but its implementation timetable must now be read as amended by Regulation (EU) 2026/1744, which entered into force on 27 July 2026 [13,49]. Under the amended Article 113, the high-risk requirements in Chapter III, Sections 1–3 apply from 2 December 2027 to systems classified under Article 6(2)/Annex III and from 2 August 2028 to systems classified under Article 6(1)/Annex I; the latter category is particularly relevant when an AI system is itself, or is a safety component of, a product covered by the Annex I product-safety legislation [49]. The FDA’s January 2025 lifecycle document for AI-enabled device software functions remains draft, non-binding guidance [14]. It should be distinguished from the FDA’s August 2025 final guidance on Predetermined Change Control Plans (PCCPs), which addresses planned post-authorization modifications to AI-enabled device software functions [50]. In the United States, 45 C.F.R. §92.210 prohibits discrimination through patient-care decision-support tools and requires covered entities to make reasonable efforts to identify and mitigate specified discrimination risks [15]. WHO guidance remains advisory [51]. Table 9 summarizes the correspondence between EA-BDMF stages and the selected regulatory, guidance, and reporting instruments.

Stage 4 requires particular care. Calibration parity and differential treatment are not equivalent, but a subgroup-specific threshold may still require protected-group membership at inference time and can change clinical action. EA-BDMF therefore treats subgroup-specific deployment thresholds as conditional on institutional clinical, legal, and ethical review. Where such a rule is not approved, the model should retain a shared threshold and report the residual subgroup disparity explicitly.

## 6. Discussion

This section interprets the review findings from both technical and healthcare-implementation perspectives. It first discusses the significance of demographic disparities across diagnostic-AI applications and the implications of the observed bias-source distribution. It then examines the scope and limits of the mitigation evidence, including cross-study comparability and intersectional fairness. The section concludes by considering the implications of EA-BDMF for healthcare delivery, organizational governance, technical assurance, and post-deployment accountability, together with the evidence required for prospective validation.

### 6.1. Principal Findings

Three findings are most consequential. First, demographic disparity in diagnostic AI is not confined to a single modality or protected attribute. The reviewed chest-radiograph literature reports TPR gaps from 0.062 to 0.281 across sex, age, and race, while dermatology evidence reports a 0.07 AUROC gap between lighter and darker Fitzpatrick groups [6,9]. These values should not be collapsed into a common magnitude, but their direction and recurrence across independent studies establish a persistent equity concern.

Second, the review identifies data representation as the most frequently reported source of bias, but the taxonomy should not be interpreted causally. The predominance of representation-related reports is plausible given the composition of widely used benchmarks—including single-center radiography cohorts and dermatology datasets with limited tonal diversity—yet the literature does not support a decomposition of observed disparity into causal percentages [7,27].

Third, reporting practice is itself a structural limitation on evidence accumulation. Item-level FRCS-10 results show that uncertainty, threshold specification, demographic-metadata provenance, intersectional analysis, and post-deployment monitoring are frequently absent. These frequencies characterize reporting completeness and do not constitute a validated overall quality measure. The estimand-compatibility assessment demonstrates a separate problem: even well-reported studies cannot be pooled when they define “disparity” using fundamentally different statistical quantities. Future fairness studies should therefore state the estimand of every reported disparity explicitly, in addition to reporting subgroup sizes, uncertainty, and thresholds.

### 6.2. Implications for Mitigation Design

The literature supports several mitigation families, but not a numerical ranking across them. The 74.7% reduction reported for prevalence-aware oversampling and the 10.6% reduction reported for synthetic augmentation are directly comparable only because they originate from the same study and metric [3]. Results from adversarial debiasing, adapter retraining, and other approaches use different cohorts and endpoints [22,33,47]. A combined EA-BDMF configuration may be complementary because its stages target different mechanisms, but that proposition remains empirical rather than established.

The fairness–accuracy relationship should be treated similarly. Clinical chest-radiograph benchmarking shows that fairness-improving methods can trade parity against subgroup performance, and that simple data balancing can be competitive with more elaborate interventions under some fairness definitions [36]. The reviewed corpus does not provide a common set of paired measurements from which a transferable optimum for λ can be estimated. Accordingly, we make no generic dose–response claim: λ and the associated task-performance cost must be estimated for the specific dataset, protected attributes, and fairness criterion on held-out data.

Intersectionality remains a further limitation of single-attribute correction. Foundation-model evaluations report particularly severe underdiagnosis at protected-attribute intersections [4]. Concurrent multi-attribute objectives are therefore preferable when sample size permits, but small intersectional cells increase uncertainty and should not be hidden by aggregate fairness scores [34,35]. LED inherits this limitation: an unenumerated intersection is invisible to the accounting system.

### 6.3. Implications for Healthcare Delivery and Governance

For healthcare organizations, aggregate AUROC should not be treated as sufficient evidence of equitable diagnostic performance across a heterogeneous population. Subgroup discrimination, calibration, uncertainty, threshold policy, and the provenance of demographic variables should be reviewed before deployment and monitored thereafter [1,12]. These requirements have implications for clinical governance as well as model engineering: responsibility for approving thresholds, interpreting residual disparity, responding to drift, and documenting remediation should be assigned explicitly rather than left to ad hoc technical decisions.

For developers, the evidence supports a staged engineering process: document the data before optimizing the model, apply data-level corrections where justified, evaluate fairness-constrained learning under a pre-specified metric, calibrate on disjoint data, and deploy only with a monitoring procedure that has an explicit error-control policy. For clinical teams, the same process provides a structured basis for deciding whether subgroup differences are clinically tolerable, whether additional validation is required, and whether a model should be paused or reassessed when equity-related performance deteriorates.

EA-BDMF’s principal governance function is traceability. The process-stage artifacts and model-bearing checkpoint certificates make explicit which evidence and decisions support each audit point. LED provides a compact summary of tolerance exceedance, while the underlying subgroup metrics and tolerances remain primary. The traceability matrix also makes coverage gaps visible, including the limited mitigation evidence for label and annotation bias in the reviewed diagnostic-AI corpus. EA-BDMF can therefore serve as a governance scaffold for aligning technical assurance, clinical oversight, and longitudinal monitoring while preserving visibility into unresolved equity risks.

## 7. Limitations and Empirical Validation Agenda

This review has methodological, evidentiary, and framework-level limitations that constrain the strength and generalizability of its conclusions. These limitations also define the principal priorities for prospective validation of EA-BDMF.

### 7.1. Limitations

The review was not prospectively registered, and no formal protocol was publicly deposited before screening. Eligibility and synthesis decisions therefore cannot be compared with a prospectively time-stamped analysis plan. In addition, title/abstract screening, data extraction, and the review-specific methodological appraisal were not independently duplicated. Although full-text eligibility was independently assessed by two assessors and FRCS-10 scoring was independently verified, single-assessor screening, extraction, and appraisal may have increased the risk of selection, coding, or judgment error.

FRCS-10 is a descriptive reporting-completeness index rather than a validated measurement instrument. Its binary items and equal weighting are pragmatic, and the observed mean of 4.6/10 should not be interpreted as a validated measure of methodological quality. Similarly, the Low/Moderate/High classification is a review-specific overall methodological appraisal. PROBAST concepts informed appraisal of primary prediction-model evidence, but formal PROBAST was not applied uniformly to secondary or contextual records. Accordingly, neither FRCS-10 nor the overall appraisal was used to determine study inclusion or quantitative-synthesis eligibility.

The evidence base limits both external validity and cross-study inference. Two of the 87 included records were available only as preprints. The exclusion criterion for exclusively proprietary evidence reduced representation of commercial and locally deployed systems. The corpus also included secondary/contextual evidence, while the quantitative evidence base was narrower and strongly weighted toward medical imaging, particularly chest radiography and dermatology. Structured EHR prediction tasks and non-U.S. settings were represented less extensively. Reported disparity estimates were not independently recomputed from patient-level predictions, and no formal small-study-effect analysis, publication-bias test, or certainty-of-evidence framework was applied because the candidate studies did not estimate a common effect-size parameter. These features limit the strength and generalizability of cross-study conclusions.

EA-BDMF has not been evaluated end to end. Its proposed stage complementarity is therefore inferred from separately reported component evidence rather than demonstrated as a pipeline effect. Likewise, LED and its checkpoint certificates provide an explicit accounting mechanism but have not been shown to predict clinical harm, clinical utility, deployment outcomes, or regulatory acceptability. The worked example in Section 5.3 illustrates calculability only. The regulatory mapping is descriptive rather than a compliance determination, and the proposed label-quality remediation gate has not been prospectively evaluated. More broadly, the framework has not yet been assessed as an organizational intervention, including its effects on workflow, implementation burden, governance processes, or patient outcomes.

### 7.2. Empirical Validation Priorities

The primary next step is a prospective, common-harness evaluation of the full framework. Such work should

- Evaluate baseline and EA-BDMF variants using patient-disjoint train, validation, calibration, and test partitions, with fixed seeds and pre-specified analysis plans;
- Report subgroup discrimination, TPR/FNR, calibration, uncertainty, and intersectional performance jointly with overall predictive accuracy;
- Pre-specify fairness-regularization, calibration, and threshold selection procedures and evaluate them only on held-out test data;
- Estimate LED trajectories prospectively and assess their sensitivity to checkpoint tolerances, weights, and monitoring policies;
- Evaluate post-deployment monitoring using statistically valid sequential procedures [39,40];
- Extend validation beyond medical imaging to structured EHR tasks, non-U.S. cohorts, and, where feasible, proprietary or locally deployed systems [52];
- Evaluate label-quality remediation strategies before downstream fairness optimization;
- Externally validate FRCS-10 and develop certainty-of-evidence approaches suitable for heterogeneous fairness estimands.

Any deployment study should obtain the required clinical, legal, and ethical approvals. The regulatory mapping presented here is descriptive and should not be interpreted as legal advice.

## 8. Conclusions

This systematic review shows that demographic performance disparities are recurrent in AI-based diagnostic models, but the available literature does not support a single pooled disparity estimate. The obstacle is not merely incomplete reporting: the most plausible candidate studies define disparity using incompatible estimands. Source-native evidence nevertheless identifies clinically relevant subgroup differences, including TPR gaps of 0.062–0.281 across sex, age, and race in chest radiography and a reported 0.07 AUROC gap across Fitzpatrick skin-tone groups in dermatology.

The review also identifies two structural deficits with direct implications for healthcare implementation. Reporting completeness is low for several elements required for reproducibility and lifecycle governance, particularly uncertainty, threshold specification, demographic-metadata provenance, intersectional analysis, and post-deployment monitoring. In parallel, label and annotation bias represents a substantial share of coded bias reports but is not repaired by the principal mitigation families represented in the reviewed diagnostic-AI fairness corpus. These gaps limit the ability of healthcare organizations to determine whether a fairness claim is reproducible, transferable, and sustainable after deployment.

EA-BDMF translates these findings into an auditable healthcare lifecycle specification. Its five stages connect data audit, prevalence-aware preprocessing, fairness-constrained learning, calibration and threshold governance, and post-deployment monitoring. LED provides a transparent tolerance-exceedance accounting construct, the traceability matrix makes mitigation coverage and gaps explicit, and FRCS-10 provides a descriptive account of reporting completeness. The complete framework remains to be validated end to end, including its clinical, organizational, and predictive utility. The framework shifts the emphasis from isolated technical debiasing toward continuous fairness governance: who measures disparity, which thresholds are acceptable, what evidence supports deployment, and how organizations respond when equity-related performance changes over time. The next evidentiary step is to evaluate the full framework under a common experimental and implementation harness, with patient-disjoint data, explicit estimands, calibrated uncertainty, intersectional analysis, prospective monitoring, structured EHR and proprietary-system evaluation where feasible, and assessment of real-world workflow and governance feasibility.

## Figures and Tables

**Table 1:** Publicly accessible datasets represented in the synthesized literature.

Dataset	Modality	Size Reported by Descriptor	Demographic Attributes	Access Model
MIMIC-CXR	Chest radiographs	377,110 images; 227,835 studies; 65,379 patients	Sex, age, race, ethnicity via MIMIC linkage	PhysioNet; credentialed access and data-use agreement [42]
CheXpert	Chest radiographs	224,316 images (approximately 65,240 patients)	Sex, age, race	Stanford ML Group; registration-based release [43]
DDI	Clinical skin images	656 biopsy-confirmed images	Fitzpatrick skin type I–VI	Stanford AIMI repository [8,28]
NIH ChestX-ray8	Chest radiographs	108,948 frontal-view images; 32,717 patients	Age, sex	NIH Clinical Center public release [44]
PadChest	Chest radiographs	168,861 images; 109,931 studies	Age, sex	Public repository, Spain [45]

**Table 2:** Taxonomy of bias sources coded across the 87 included studies.

Bias Source	Representative Mechanism	Share of Coded Instances
Data representation [7,27]	Institutional or geographic sampling skew; racial or skin-tone underrepresentation	38%
Algorithmic design [4,6]	Shortcut learning; latent encoding of protected attributes	21%
Label and annotation [29,46]	Annotator bias; proxy-outcome mislabeling; invalid annotation scale	19%
Deployment and feedback loop [1]	Automation bias; alert fatigue; distribution shift	12%
Evaluation and reporting gap [1]	Missing subgroup-stratified evaluation or insufficient documentation	10%

**Table 3:** FRCS-10 item definitions and reporting completeness.

No.	Reporting Element	Records (of 87)	%
1	Protected attributes explicitly enumerated	87	100
2	Demographic-metadata provenance documented (source, coding scheme, missingness)	26	30
3	Sample size reported for every analyzed subgroup	72	83
4	Subgroup performance reported with uncertainty (CI or SE)	38	44
5	Fairness metric formally defined rather than only named	55	63
6	Decision threshold and selection procedure reported	31	36
7	Patient-level rather than image-level splitting explicitly confirmed	41	47
8	Intersectional analysis across at least two attributes reported	19	22
9	Mitigation effect and accuracy cost reported on the same cohort	22	25
10	Post-deployment or temporal fairness monitoring plan described	7	8
Descriptive item-total summary: mean 4.6/10; median 4 (IQR 3–6);		–	–
5 studies reported ≥8 items; none reported all 10			

**Table 4:** Baseline demographic disparities reported in independent studies.

Dataset/Evidence Source	Subgroup Comparison	Metric	Reported Value	Source
CheXpert	Sex (M vs. F)	TPR gap	0.062	[6]
MIMIC-CXR	Sex (M vs. F)	TPR gap	0.072	[6]
CheXpert	Age (young vs. old)	TPR gap	0.272	[6]
MIMIC-CXR	Age (young vs. old)	TPR gap	0.245	[6]
CheXpert	Race (White vs. Black)	TPR gap	0.226	[6]
MIMIC-CXR	Race (White vs. Black)	TPR gap	0.281	[6]
Pooled dermatology cohorts	Fitzpatrick I–III vs. IV–VI	AUROC gap	0.070	[9]

**Table 5:** Source-attributed evidence for major mitigation strategies.

Source	Intervention	Source-Defined Metric/Cohort	Reported Effect and Comparability
Jackson et al. [3]	Prevalence-based oversampling	Race-associated FNR difference; MIMIC-CXR	74.7% relative disparity reduction; post-mitigation AUC 95% CI 0.810–0.819. Directly comparable only with the synthetic-augmentation row below.
Jackson et al. [3]	Synthetic image augmentation	Same cohort and metric	10.6% relative disparity reduction; post-mitigation AUC 95% CI 0.817–0.821.
Mottez et al. [22]	Lightweight adapter retraining with XGBoost, with/without active learning	Sex, age, and race across CheXpert and MIMIC-CXR	Bias reduction reported across variants; no single reduction scalar permits comparison with Jackson et al.
Mir et al. [33]	Adversarial/fairness-aware training (systematic review)	Heterogeneous primary studies	Directional evidence of disparity reduction; no pooled magnitude reported.
Luo et al. [47]	Council-guided adaptive agreement	Benchmark accuracy under induced bias	Improvement reported on the benchmark scale; not a subgroup-disparity effect size.

**Table 6:** Estimand-compatibility assessment of five candidate studies considered for quantitative synthesis.

Study	Estimand Defined by Source	Evaluation Data	Reason Not Poolable on a Common Scale
Seyyed-Kalantari et al. [26]	False-positive rate of the binarized “no finding” label (underdiagnosis rate)	MIMIC-CXR, CheXpert, NIH ChestX-ray14	A rate on a derived label, not a subgroup performance-gap estimand.
Kurian et al. [6]	TPR gaps between demographic subgroups	CheXpert and MIMIC-CXR	Multiple subgroup- and dataset-specific gaps; no single study-level effect.
Jackson et al. [3]	Race-associated FNR difference and relative mitigation reduction	MIMIC-CXR	Different metric and reporting scale from TPR gaps.
Yang et al. [4]	Thresholded underdiagnosis/FNR disparities across demographic groups; rank-based tests for selected model–radiologist comparisons	Five public chest-radiograph datasets	Multiple dataset-, pathology-, and subgroup-specific disparities; no single study-level effect directly commensurable with the other candidates.
Luo et al. [47]	Benchmark accuracy under seven induced-bias scenarios	Four medical-imaging datasets	Overall model performance, not a subgroup-disparity estimand.

**Table 7:** EA-BDMF process stages, targeted bias sources, methodological actions, and corresponding audit outputs.

Stage	Bias Source Addressed	Methodological Action	Audit Artifact/Checkpoint Output
1. Data audit	Data representation; evaluation/reporting	Metadata completeness, subgroup prevalence, label/annotation provenance [1,11]	Missingness and prevalence tables; annotation scheme; declared tolerances; no model-performance LED residual
2. Preprocessing	Data representation	Prevalence-aware resampling/reweighting; subgroup-restricted augmentation [3]	Sampling/weight policy; post-processing prevalence summary; checkpoint $c_{2}$ after retraining
3. Training	Algorithmic design	Adversarial or multi-attribute fairness objective [33,34]	λ; adversary specification; checkpoint $c_{3}$ residual
4. Calibration/governance	Algorithmic design; evaluation	Calibration plus shared or governance-approved subgroup thresholds [36,37]	Calibration split; reliability analysis; threshold table; approval decision; checkpoint $c_{4}$ residual
5. Monitoring	Deployment/feedback	Sequential fairness and calibration-drift surveillance [38,39,40]	Monitoring window; error budget; LED trace; remediation log

**Table 8:** Bias Provenance–Mitigation Traceability Matrix.

Coded Bias Source	Acting Stage(s)	Mitigation Mechanism/Evidence Class	Verifiable Artifact
Data representation (38%)	1, 2	Metadata audit and prevalence-aware preprocessing; direct empirical support [3,11]	Pre/post subgroup prevalence; sampling or weighting policy
Algorithmic design (21%)	3, 4	Fairness-constrained learning and calibration/threshold governance; direct or component-level support [22,33,34]	Checkpoint residuals; λ; calibration and threshold records
Label/annotation (19%)	1 (audit/remediation gate)	Provenance auditing plus documented re-annotation/adjudication or label-sensitivity decision when indicated; no preferred correction method was validated in this corpus [29,30]	Label provenance; annotation scale; annotator agreement; remediation/sensitivity record
Deployment/feedback (12%)	5	Sequential drift and calibration surveillance; indirect clinical-fairness deployment evidence [39,40]	Monitoring log; LED trace; remediation events
Evaluation/reporting (10%)	1–5 instrumentation	Provenance logging, stage certificates, FRCS-10 [10,38]	Completed stage certificates and reporting record

**Table 9:** Mapping of EA-BDMF stages to selected regulatory and reporting instruments (EU AI Act provisions are read as amended by Regulation (EU) 2026/1744).

EA-BDMF Stage	EU AI Act, as Amended	FDA Lifecycle Guidance	Other Instruments
1. Data audit	Art. 10 data governance; Art. 11 technical documentation	Representativeness of development and validation data	STANDING Together [11]
2. Preprocessing	Art. 10(2) bias examination/mitigation; Art. 9 risk management	Data-management and bias-control measures	45 C.F.R. §92.210 [15]
3. Training	Art. 15 accuracy, robustness, cybersecurity	Model development and performance characterization	TRIPOD+AI [10]
4. Calibration/governance	Arts. 13–14 transparency and human oversight	Subgroup performance evaluation and labeling/disclosure	45 C.F.R. §92.210; WHO ethics guidance [15,51]
5. Monitoring	Art. 72 post-market monitoring; Art. 9 continuous risk management	Post-market performance monitoring; PCCP governance for planned modifications [50]	DECIDE-AI and statistically valid monitoring [25,39]

## Data Availability

No new patient-level data were generated in this study. The public benchmark datasets discussed in this review remain available from their original custodians under the stated access conditions. Review-level supporting materials are provided in Supplementary Tables S1–S4 and Files S1 and S2, including the PRISMA 2020 checklist; the characteristics of all 87 included records; study-level overall methodological appraisal and FRCS-10 totals; screening dispositions for all 187 reports; publication-status and quantitative-eligibility summaries; bias-source coding rules; PRISMA flow details; and the illustrative EA-BDMF/LED worked example. These materials contain no patient-level records.

## Supplementary Materials

The following supporting information can be downloaded at https://www.mdpi.com/article/10.3390/healthcare14193293/s1: Supplementary File S1, PRISMA 2020 checklist; Supplementary Table S1, study-level overall methodological appraisal and FRCS-10 totals for all 87 included records, with PROBAST-informed mapping and detailed empirical examples; Supplementary Table S2, characteristics of all 87 included records and their evidence roles; Supplementary Table S3, screening dispositions for all 187 reports, including 24 reports not retrieved and 163 reports excluded after eligibility assessment; Supplementary Table S4, bias-source coding rules, publication-status summary, PRISMA flow details, evidence-role summary, and quantitative-eligibility criteria; and Supplementary File S2, an illustrative EA-BDMF/LED worked example.
