# Supplementary material for: Algorithmic Bias in AI-Based Diagnostic Models: A Systematic Review and Lifecycle Framework for Fairness Auditing and Governance in Healthcare
Source: Healthcare (Basel). 2026 Oct 2;14(19):3293. doi: 10.3390/healthcare14193293 (PMC13665230; doi:10.3390/healthcare14193293)
Supplement: Supplementary file 1 [file healthcare-14-03293-s001.zip › Supplementary_File_S1_PRISMA_2020_Checklist_Submission.pdf]

PRISMA 2020 Checklist

Manuscript: Algorithmic Bias in AI-Based Diagnostic Models: A Systematic Review and Lifecycle Framework for Fairness Auditing and Governance in Healthcare

Supplementary File S1

Section and Topic	Item #	Checklist item	Location where item is reported
**TITLE**<br>**Title**	1	Identify the report as a systematic review.	Title page; manuscript type is identified as “Systematic Review”.
**ABSTRACT**<br>**Abstract**	2	See the PRISMA 2020 for Abstracts checklist.	Structured Abstract (Background, Methods, Results, Conclusions).
**INTRODUCTION**<br>**Rationale**	3	Describe the rationale for the review in the context of existing knowledge.	Section 1, Introduction, paragraphs 1–4.
**INTRODUCTION**<br>**Objectives**	4	Provide an explicit statement of the objective(s) or question(s) the review addresses.	Section 1, final paragraph (four stated objectives).
**METHODS**<br>**Eligibility criteria**	5	Specify the inclusion and exclusion criteria for the review and how studies were grouped for the syntheses.	Section 3.1, Review Design, Eligibility, and Search Strategy.
**METHODS**<br>**Information sources**	6	Specify all databases, registers, websites, organisations, reference lists and other sources searched or consulted to identify studies. Specify the date when each source was last searched or consulted.	Section 3.1: PubMed/MEDLINE, IEEE Xplore, ACM Digital Library, Scopus, Web of Science, and arXiv; searched 20 August 2026.
**METHODS**<br>**Search strategy**	7	Present the full search strategies for all databases, registers and websites, including any filters and limits used.	Section 3.1: core Boolean search expression and statement that syntax was adapted to each database, including controlled-vocabulary terms where available.
**METHODS**<br>**Selection process**	8	Specify the methods used to decide whether a study met the inclusion criteria of the review, including how many reviewers screened each record and each report retrieved, whether they worked independently, and if applicable, details of automation tools used in the process.	Section 3.2: title/abstract screening by the author; 274 reports sought for retrieval; 24 reports not retrieved; independent eligibility assessment of 250 retrieved full texts by the author and an external assessor; third assessor for unresolved disagreements; Cohen’s κ reported.
**METHODS**<br>**Data collection process**	9	Specify the methods used to collect data from reports, including how many reviewers collected data from each report, whether they worked independently, any processes for obtaining or confirming data from study investigators, and if applicable, details of automation tools used in the process.	Section 3.2: predefined structured extraction form; 87-record study-characteristics register and 87-row appraisal/FRCS table supplied in Supplementary Tables S2-S3; extraction by the author and not independently duplicated.
**METHODS**<br>**Data items**	10a	List and define all outcomes for which data were sought. Specify whether all results that were compatible with each outcome domain in each study were sought, and if not, the methods used to decide which results to collect.	Sections 3.2–3.4: subgroup-stratified performance, fairness metrics, mitigation effects, uncertainty, thresholds; Eq. (1) defines EOD magnitude.
**METHODS**<br>**Data items**	10b	List and define all other variables for which data were sought. Describe any assumptions made about any missing or unclear information.	Section 3.2: study design, dataset, demographic attributes, subgroup sample sizes, thresholds, mitigation strategy, uncertainty and effect estimates; Section 3.4 describes source-native treatment of non-comparable measures.
**METHODS**<br>**Study risk of bias assessment**	11	Specify the methods used to assess risk of bias in the included studies, including details of the tool(s) used, how many reviewers assessed each study and whether they worked independently, and if applicable, details of automation tools used in the process.	Section 3.2: review-specific overall methodological appraisal for all 87 records; PROBAST-informed Participants, Predictors, Outcome and Analysis concepts applied to primary prediction-model evidence; original signaling questions not applied verbatim. Supplementary Table S2 provides all 87 overall appraisals/FRCS totals, mapping, and detailed empirical examples.
**METHODS**<br>**Effect measures**	12	Specify for each outcome the effect measure(s) used in the synthesis or presentation of results.	Section 3.4 and Eq. (1): source-native measures retained; EOD expressed as absolute TPR gap; AUROC, FNR/TPR and other source-defined measures reported without forced conversion.

Section and Topic	Item #	Checklist item	Location where item is reported
**METHODS**<br>**Synthesis methods**	13a	Describe the processes used to decide which studies were eligible for each synthesis.	Section 3.5: among the 87 included records, 46 underwent structured quantitative extraction (41 non-secondary and 5 secondary/contextual). A separate 12-record reporting-completeness screen and five-study estimand-compatibility assessment are described; Supplementary Table S5 presents the evidence-role and quantitative-eligibility details.
**METHODS**<br>**Synthesis methods**	13b	Describe any methods required to prepare the data for presentation or synthesis, such as handling of missing summary statistics, or data conversions.	Section 3.4: incompatible TPR gaps, FNR differences, AUROC differences, rank-based tests and benchmark accuracy were not transformed to a common effect-size scale.
**METHODS**<br>**Synthesis methods**	13c	Describe any methods used to tabulate or visually display results of individual studies and syntheses.	Sections 4.1–4.5; Figures 2–3; Tables 2–6.
**METHODS**<br>**Synthesis methods**	13d	Describe any methods used to synthesize results and provide a rationale for the choice(s). If meta-analysis was performed, describe the model(s), method(s) to identify the presence and extent of statistical heterogeneity, and software package(s) used.	Sections 3.4 and 4.5: thematic/source-native synthesis plus explicit estimand-compatibility audit; no meta-analysis was performed because the candidate effect measures were not commensurable.
**METHODS**<br>**Synthesis methods**	13e	Describe any methods used to explore possible causes of heterogeneity among study results.	Not applicable: no common-effect statistical synthesis or meta-analysis was performed; rationale is given in Sections 3.4 and 4.5.
**METHODS**<br>**Synthesis methods**	13f	Describe any sensitivity analyses conducted to assess robustness of the synthesized results.	Not applicable: no pooled statistical synthesis was conducted.
**METHODS**<br>**Reporting bias assessment**	14	Describe any methods used to assess risk of bias due to missing results in a synthesis (arising from reporting biases).	Not applicable to a pooled synthesis: no formal small-study-effect or reporting-bias analysis was performed because there was no defensible common effect-size set; limitation discussed in Section 7.1.
**METHODS**<br>**Certainty assessment**	15	Describe any methods used to assess certainty (or confidence) in the body of evidence for an outcome.	Not applicable: no formal certainty-of-evidence framework was applied because the review synthesized heterogeneous diagnostic-fairness estimands rather than a common intervention effect.
**RESULTS**<br>**Study selection**	16a	Describe the results of the search and selection process, from the number of records identified in the search to the number of studies included in the review, ideally using a flow diagram.	Section 3.2 and Figure 1: 1,842 records identified; 527 duplicates removed; 1,315 records screened; 1,041 excluded at title/abstract; 274 reports sought; 24 not retrieved; 250 reports assessed for eligibility; 163 excluded after assessment; 87 records included; 46 underwent structured quantitative extraction.
**RESULTS**<br>**Study selection**	16b	Cite studies that might appear to meet the inclusion criteria, but which were excluded, and explain why they were excluded.	Supplementary Table S4 provides the complete FT001-FT187 screening-disposition table. It distinguishes 24 reports not retrieved from 163 reports excluded after eligibility assessment and records the primary disposition reason for every entry.
**RESULTS**<br>**Study characteristics**	17	Cite each included study and present its characteristics.	Supplementary Table S3 presents characteristics for all 87 included records, including citation, study design, clinical task/modality, dataset/cohort, protected attributes, evidence role, and publication status.
**RESULTS**<br>**Risk of bias in studies**	18	Present assessments of risk of bias for each included study.	Supplementary Table S2 provides the study-specific review-level overall methodological appraisal and FRCS-10 total for all 87 records. It also maps the appraisal logic to PROBAST/PROBAST+AI concepts and gives detailed domain-level examples for the principal empirical quantitative studies; the 87-row Low/Moderate/High column is not presented as formal PROBAST for secondary/contextual records.
**RESULTS**<br>**Results of individual studies**	19	For all outcomes, present, for each study: (a) summary statistics for each group (where appropriate) and (b) an effect estimate and its precision, ideally using structured tables or plots.	Sections 4.3–4.5; Figure 3; Tables 4–6 present source-attributed subgroup disparities, mitigation effects and estimand definitions for the principal quantitative studies.
**RESULTS**<br>**Results of syntheses**	20a	For each synthesis, briefly summarise the characteristics and risk of bias among contributing studies.	Sections 4.3–4.5, together with the aggregate risk-of-bias appraisal in Section 3.2.

Section and Topic	Item #	Checklist item	Location where item is reported
**RESULTS**<br>**Results of syntheses**	20b	Present results of all statistical syntheses conducted. If meta-analysis was done, present for each the summary estimate and its precision and measures of statistical heterogeneity. If comparing groups, describe the direction of the effect.	Section 4.5: no pooled statistical synthesis was conducted; the estimand audit explains why a defensible pooled effect could not be computed.
**RESULTS**<br>**Results of syntheses**	20c	Present results of all investigations of possible causes of heterogeneity among study results.	Not applicable: no common-effect meta-analysis was undertaken.
**RESULTS**<br>**Results of syntheses**	20d	Present results of all sensitivity analyses conducted to assess the robustness of the synthesized results.	Not applicable: no pooled statistical synthesis was undertaken.
**RESULTS**<br>**Reporting biases**	21	Present assessments of risk of bias due to missing results (arising from reporting biases) for each synthesis assessed.	Not applicable: no formal reporting-bias assessment was undertaken because there was no defensible common effect-size set; this limitation is stated in Section 7.1.
**RESULTS**<br>**Certainty of evidence**	22	Present assessments of certainty (or confidence) in the body of evidence for each outcome assessed.	Not applicable: no formal certainty-of-evidence assessment was performed.
**DISCUSSION**<br>**Discussion**	23a	Provide a general interpretation of the results in the context of other evidence.	Section 6.1, Principal Findings.
**DISCUSSION**<br>**Discussion**	23b	Discuss any limitations of the evidence included in the review.	Section 7.1, Limitations.
**DISCUSSION**<br>**Discussion**	23c	Discuss any limitations of the review processes used.	Section 7.1, including limitations related to the evidence base, publication/selection bias, and absence of a common effect-size set.
**DISCUSSION**<br>**Discussion**	23d	Discuss implications of the results for practice, policy, and future research.	Sections 6.2–6.3 and 7.2.
**OTHER INFORMATION**<br>**Registration and protocol**	24a	Provide registration information for the review, including register name and registration number, or state that the review was not registered.	Section 3.1: statement that the review was not prospectively registered in PROSPERO or another public registry.
**OTHER INFORMATION**<br>**Registration and protocol**	24b	Indicate where the review protocol can be accessed, or state that a protocol was not prepared.	Section 3.1: no separate publicly accessible protocol is reported.
**OTHER INFORMATION**<br>**Registration and protocol**	24c	Describe and explain any amendments to information provided at registration or in the protocol.	Not applicable: the review was not prospectively registered; no registered protocol amendments apply.
**OTHER INFORMATION**<br>**Support**	25	Describe sources of financial or non-financial support for the review, and the role of the funders or sponsors in the review.	Funding statement: "This research received no external funding."
**OTHER INFORMATION**<br>**Competing interests**	26	Declare any competing interests of review authors.	Conflicts of Interest statement.
**OTHER INFORMATION**<br>**Availability of data, code and other materials**	27	Report which of the following are publicly available and where they can be found: template data collection forms; data extracted from included studies; data used for all analyses; analytic code; any other materials used in the review.	Supplementary Files/Tables S1-S6 provide the PRISMA checklist; study-level overall methodological appraisal and FRCS-10 totals; characteristics of all 87 included records; the FT001-FT187 screening-disposition table; bias-coding, publication-status, PRISMA-flow, evidence-role and quantitative-eligibility summaries; and an illustrative EA-BDMF/LED worked example. No patient-level data are included.

Source: Page MJ, McKenzie JE, Bossuyt PM, Boutron I, Hoffmann TC, Mulrow CD, et al. The PRISMA 2020 statement: an updated guideline for reporting systematic reviews. BMJ. 2021;372:n71. doi:10.1136/bmj.n71. PRISMA 2020 checklist licensed under CC BY 4.0.
