# Supplementary material for: Algorithmic Bias in AI-Based Diagnostic Models: A Systematic Review and Lifecycle Framework for Fairness Auditing and Governance in Healthcare
Source: Healthcare (Basel). 2026 Oct 2;14(19):3293. doi: 10.3390/healthcare14193293 (PMC13665230; doi:10.3390/healthcare14193293)
Supplement: Supplementary file 1 [file healthcare-14-03293-s001.zip › Supplementary_File_S2_EABDMF_LED_Worked_Example_Submission.pdf]

Supplementary File S2

Illustrative EA-BDMF / Lifecycle Equity Debt Worked Example

This example demonstrates how the LED accounting layer can be calculated from a source-reported disparity and mitigation effect. It is not an empirical validation of the complete EA-BDMF workflow.

Source-reported inputs

**Source**	Jackson et al. (2025), Enhancement of Fairness in AI for Chest X-Ray Classification
**Baseline race-associated FNR disparity**	Approximately 0.084
**Reported relative reduction after prevalence-based oversampling**	74.7%
**Illustrative post-mitigation disparity**	0.084 × (1 − 0.747) = 0.021252 ≈ 0.0213

Illustrative governance assumptions

For demonstration only, assume a pre-declared race-disparity tolerance $\varepsilon = 0.05$ and equal checkpoint weights $w_0 = w_2 = 0.5$. These values are hypothetical governance parameters and are not derived from Jackson et al.

Checkpoint calculation

$$\mathrm{d}_0 = \max\{0, 0.084 / 0.05 - 1\} = 0.68$$

$$\mathrm{d}_2 = \max\{0, 0.021252 / 0.05 - 1\} = 0$$

$$\mathrm{LED} = 0.5 \times 0.68 + 0.5 \times 0 = 0.34$$

Interpretation

- The example shows that the accounting construct is calculable and that a source-reported reduction in subgroup disparity can be translated into a lower tolerance-exceedance residual when the same metric and pre-declared tolerance are used.
- The result does not establish that $\varepsilon = 0.05$ is clinically appropriate, that equal checkpoint weights are optimal, or that LED predicts patient harm, clinical utility, deployment safety, or regulatory compliance.
- A prospective validation would need fixed subgroup definitions, patient-disjoint audit data, uncertainty estimates, clinically justified tolerances, pre-specified checkpoint weights, and outcome-linked monitoring.

Scope of interpretation

This worked example is intended only to demonstrate the calculability and interpretation of the LED accounting construct. It should not be interpreted as empirical validation of EA-BDMF, as evidence that the illustrative tolerance or checkpoint weights are clinically optimal, or as evidence that LED predicts patient harm, clinical utility, deployment safety, or regulatory compliance.
